# New Progress in Basic Research of Macrophages in the Pathogenesis and Treatment of Low Back Pain

**DOI:** 10.3389/fcell.2022.866857

**Published:** 2022-05-20

**Authors:** Miaoheng Yan, Zongmian Song, Hongwei Kou, Guowei Shang, Chunfeng Shang, Xiangrong Chen, Yanhui Ji, Deming Bao, Tian Cheng, Jinfeng Li, Xiao Lv, Hongjian Liu, Songfeng Chen

**Affiliations:** ^1^ Department of Orthopedics, The First Affiliated Hospital of Zhengzhou University, Zhengzhou, China; ^2^ Department of Orthopedics, Union Hospital, Tongji Medical College, Huazhong University of Science and Technology, Wuhan, China

**Keywords:** macrophages, inflammatory immune response, low back pain, intervertebral disc degeneration, muscle, dorsal root ganglion

## Abstract

Low back pain (LBP) is quite common in clinical practice, which can lead to long-term bed rest or even disability. It is a worldwide health problem remains to be solved. LBP can be induced or exacerbated by abnormal structure and function of spinal tissue such as intervertebral disc (IVD), dorsal root ganglion (DRG) and muscle; IVD degeneration (IVDD) is considered as the most important among all the pathogenic factors. Inflammation, immune response, mechanical load, and hypoxia etc., can induce LBP by affecting the spinal tissue, among which inflammation and immune response are the key link. Inflammation and immune response play a double-edged sword role in LBP. As the main phagocytic cells in the body, macrophages are closely related to body homeostasis and various diseases. Recent studies have shown that macrophages are the only inflammatory cells that can penetrate the closed nucleus pulposus, expressed in various structures of the IVD, and the number is positively correlated with the degree of IVDD. Moreover, macrophages play a phagocytosis role or regulate the metabolism of DRG and muscle tissues through neuro-immune mechanism, while the imbalance of macrophages polarization will lead to more inflammatory factors to chemotaxis and aggregation, forming an “inflammatory waterfall” effect similar to “positive feedback,” which greatly aggravates LBP. Regulation of macrophages migration and polarization, inhibition of inflammation and continuous activation of immune response by molecular biological technology can markedly improve the inflammatory microenvironment, and thus effectively prevent and treat LBP. Studies on macrophages and LBP were mainly focused in the last 3–5 years, attracting more and more scholars’ attention. This paper summarizes the new research progress of macrophages in the pathogenesis and treatment of LBP, aiming to provide an important clinical prevention and treatment strategy for LBP.

## Introduction

Low back pain (LBP) is extremely common and mostly manifested as lumbosacral pain with or without radicular symptom (Knezevic et al., 2021), up to 75–80% of people suffer from LBP during their lifetime at some point (Chen et al., 2017). LBP greatly affects the patients’ quality of life, and even makes patients disabled in severe cases, bringing huge socioeconomic burden. The Lancet reported that LBP ranks sixth in the global disease burden (Global Burden of Disease Study 2013 Collaborators, 2015). In 2016 alone, the United States spent $134.5 billion on the treatment of LBP and neck pain (Dieleman et al., 2020), and the indirect loss caused by LBP is even higher than the direct medical costs (Kigozi et al., 2019). With the advent of aging society, the prevalence and number of patients with LBP continue to increase globally; meanwhile, the incidence of LBP also displays a gradual increased trend in young people. It is reported that about 40% children aged from 9 to 18 years suffer from LBP (Calvo-Muñoz et al., 2013; Kędra et al., 2021).

The conventional treatment for LBP includes physical therapy, oral medicine, local injection and surgical treatment (Table 1). Generally, physical exercise therapy is the first choice, but patients’ compliance is often poor during the implementation of such therapy. Patients should be guided to actively cooperate pain treatment and alleviate fear related to pain and avoidance behavior (Vlaeyen et al., 2018). If the symptoms of LBP are not relieved or progressively worsen, oral drug is often required. The non-steroidal anti-inflammatory drugs (NSAIDs) are the commonly used drugs. The alternative drugs also include opioids, antidepressants and muscle relaxants etc. American College of Physicians guidelines consider NSAIDs or muscle relaxants to be the first choice drugs for acute or subacute LBP (Qaseem et al., 2017). For oral drugs with poor efficacy and long-term side effects such as gastrointestinal discomfort or addiction, local injection of drugs should be taken into consideration. The steroid is commonly used local injection drug, which exert analgesic effect on LBP caused by nucleus pulposus (NP) herniation, but has a poor effect on most patients with spinal stenosis or IVDD without nerve compression (Cohen et al., 2013). Following above treatment, if the LBP continues to aggravate, surgery should be actively performed. Compared with other therapies, surgical treatment can relieve pain faster and markedly improve function (Bailey et al., 2020). However, 10–40% of patients may develop into failured back surgery syndrome (FBSS), which means that patients will suffer from recurrent LBP accompanied by neurological symptoms after one or several surgeries (Chan and Peng, 2011). The non-surgical treatment only relieve the symptoms, and surgical treatment has the defects of recurrence, aggravation of adjacent segment degeneration and difficulty in restoring normal biological function of the spine. In-depth explore the precise pathogenesis of LBP is expected to provide new strategy for effective prevention and treatment of LBP, which has important scientific significance and clinical value.

**TABLE 1:** Conventional treatment options for LBP.

Treatment options	Characteristics	Disadvantage	References
Behavioural management	Advice to stay active, Patient education, Cognitive behavioural therapy; Relieve mild LBP	Poor patient self-management	Vlaeyen et al. (2018)
Physical therapy	Spinal manipulation, Diathermy therapy, Acupuncture; Have a certain effect for LBP	Small improvements	Knezevic et al. (2021)
Pharmacological treatment	Non-steroidal anti-inflammatory drugs (NSAIDs) or muscle relaxants; First choice for acute LBP	Gastrointestinal side effect	Qaseem et al. (2017)
	Antidepressants, weak opioids Second-line drug options for chronic LBP	Short-term	Knezevic et al. (2021)
	Opioids Last-line drug options for chronic LBP	Addictive potential	Knezevic et al. (2021)
Local injection	Epidural steroid injection; Reduces nerve root pain	Risk of puncture into blood vessels, infection	Cohen et al. (2013)
Surgery	Discectomy, laminectomy, lumbar fusion; Faster pain relief and functional improvement	Failed back surgery syndrome	Bailey et al. (2020)

Literatures have confirmed that IVDD and the dysfunction of IVD peripheral structures, such as paraspinal muscles, dorsal root ganglia (DRG), and facet joints, are the direct cause and core link of LBP. The IVD consists of the gelatinous NP in the central part, the inner and outer annular fibers (AF), and the upper and lower cartilage endplates (CEP). IVDD is the most important pathogenic factor of LBP. During the process of IVDD, inflammation, mechanical load and other factors could cause diminished function and decreased number of IVD cells, and degradation of proteoglycans and type II collagen, which further aggravates the damage to IVD microenvironment and results in discogenic LBP (Gilbert et al., 2016). When IVDD occurs, infiltration of sensory nerves and blood vessels into the CEP can further irritate the inflammation of the IVD, which is a crucial source of LBP. Modic et al. (1988) discovered that patients with LBP often exhibit changes in MRI signal intensity of endplates (EP) and adjacent vertebral subchondral bone. Inflammation and immune response exert an pivotal role in the pathological process of Modic changes, ultimately triggering LBP to a large extent. Additionally, the abnormal structure and function of muscle, DRG, facet joint etc., are closely related to inflammation and immune response. Macrophages migrate to the damaged area and release inflammatory factors, which cause inflammatory cascade reactions. Increased inflammatory and chemokine factors will lead to hyperalgesia. The abnormal structure and function of vertebral adjacent tissues interact with the degeneration of the IVD and inflammatory immune response to activate the “positive feedback” amplification effect, which is extremely crucial in inducing and exacerbating LBP.

Structural and functional abnormalities of the IVD and its adjacent tissues often result in LBP, which is attributed to macrophage-mediated inflammation and immune response to a certain extent. In this paper, the latest basic research progress of macrophages in the pathogenesis of LBP is summarized, in order to explore the key molecules or signal pathways that can be precisely targeted and regulated, and provide an important basis for effective clinical prevention and treatment of LBP.

### Literatures Screening Methods

**FIGURE 1:**
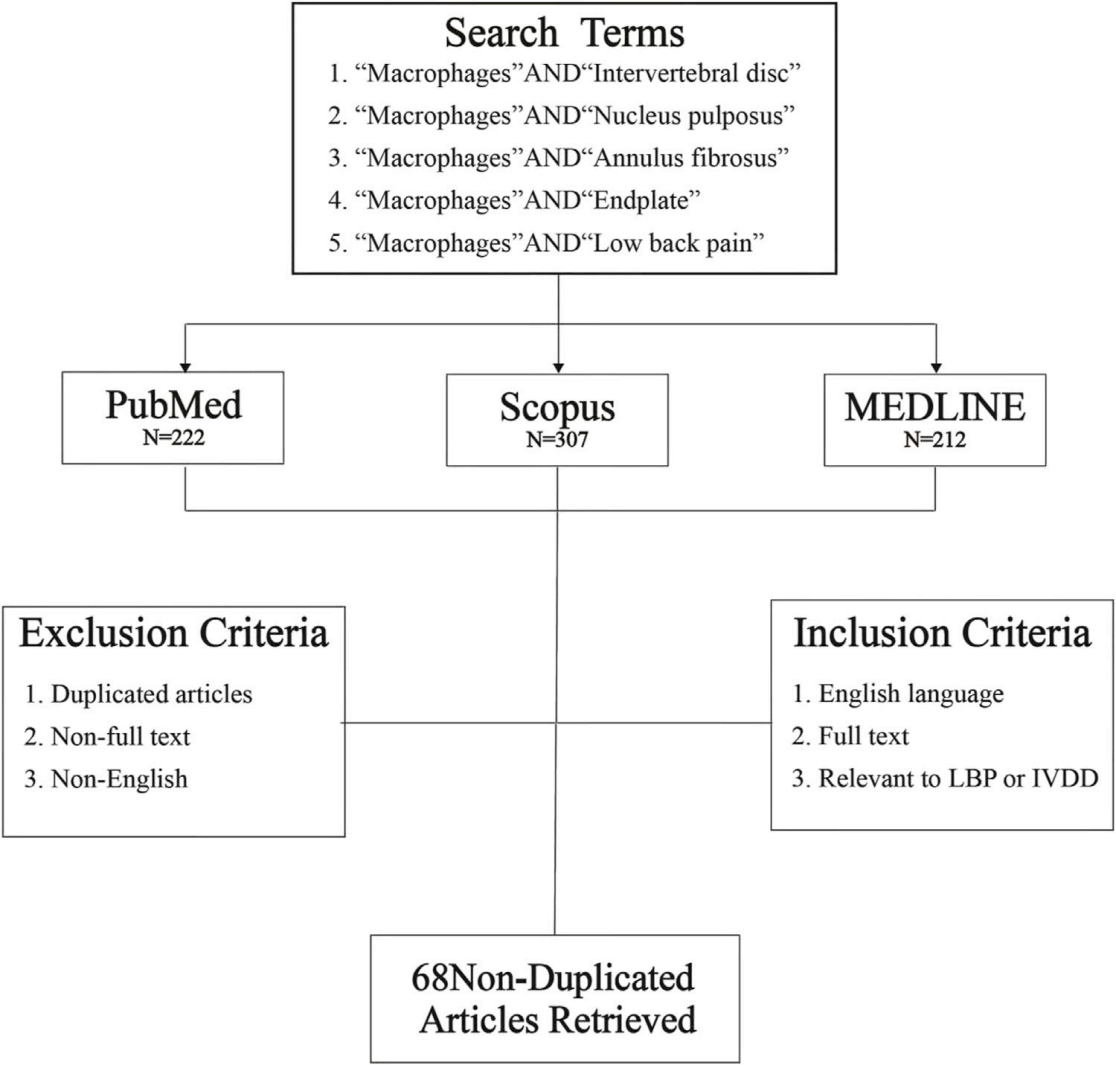
Database search flowchart. Five independent literature searches were conducted using defined search terms through three literature databases, and the identified articles were screened for exclusion or inclusion. Then, 741 articles were obtained from the initial database search; after screening, 68 non-duplicated original research articles were included in this review.

This review selected literatures from PubMed, Scopus and MEDLINE databases, all literatures were searched with the key words of “Macrophages and Intervertebral disc”, “Macrophages and Nucleus pulposus,” “Macrophages and Annulus fibrosus,” “Macrophages and Endplate” and “Macrophages and Low back pain” since January 2017. Then, 222 articles were retrieved from PubMed database, 307 articles from Scopus, and 212 articles from MEDLINE. In total, 741 references were obtained. After removal of duplicates, each article was carefully examined according to inclusion and exclusion criteria. After screening, 68 articles met inclusion criteria, including: 1. English language, 2. Full text, 3. Relevant to LBP or IVDD (Figure 1). We reviewed 68 non-duplicate original articles to elucidate the effects of macrophages polarization on IVD structures and their adjacent structures; discussed the mechanisms of macrophages-induced LBP and potential intervention molecular targets.

### Macrophages and Orthopedic Diseases Origin, Type and Function of Macrophages

Macrophages have long been considered to be important immune effector cells; they are mainly derived from adult bone marrow-derived hematopoietic stem cells (HSCs), while others are produced by embryonic hematopoietic progenitor cells (Ginhoux and Guilliams, 2016). The macrophages originate from the yolk sac and present two waves, one directly generated in the yolk sac and then distributed throughout the embryo (Hoeffel et al., 2015). Another wave is made up of yolk sac-derived myeloid-biased progenitor cells and migrate into the liver, several lineages are produced, including monocytes; and then differentiate into macrophages, myeloid progenitor cells in liver and specialize into monocytes and granulocytes, respectively (McGrath et al., 2015). Macrophages were differentiated from monocytes, which indicated that hematopoietic progenitors produced by yolk sac have the ability to differentiate into macrophages in liver (Bian et al., 2020). Macrophages exist in tissues in a stable state, participate in inflammatory reactions and produce chemokines (Brown et al., 2012). Monocytes can be divided into “inflammatory” and “resident” subpopulations before they differentiate into macrophages. Inflammatory monocytes can rapidly recruit and migrate to the region of injury or infection via C-C chemokine receptor type 2 (CCR2), C-C motif chemokine ligand 2 (CCL2), monocyte chemotactic protein-1 (MCP-1), and C-X-C motif ligand 10 (CXCL10). The resident monocytes lack CCR2 expression and could patrol the vascular system, fill normal tissues, and regulate inflammatory responses (Orozco et al., 2021). Aegerter et al. (2020) reported that influenza-induced alveolar macrophages produced more interleukin-6 (IL-6) and had better resistance to *Streptococcus pneumoniae* infection in mice. Yao et al. (2018) documented that viral infection induce long-acting memory alveolar macrophages and control bacterial infection. Macrophages have a large plastic phenotype and display distinct subtype changes and functional differences in different microenvironments.

M0 macrophages are non-activated, which can be activated in both classical activation (M1) and alternative activation (M2). Some scholars further divided M2 macrophages into M2a, M2b and M2c subtypes. M1 macrophages secrete many pro-inflammatory cytokines and have high bactericidal activity. M2a macrophages could exert anti-inflammatory effects, promote tissue remodeling and wound healing. M2b macrophages can promote tumor growth and exert immunomodulatory effects. M2c macrophages can enhance apoptotic body phagocytosis, tissue remodeling and immunosuppressive effects (Viola et al., 2019). Due to overlapping expression markers, the “M1/M2” taxonomy is currently most commonly used. The surface markers of M1 macrophages are mainly CD197 (CCR7), CD80 and CD86, which can secrete inflammatory factors such as tumor necrosis factor-α (TNF-α), IL-1β, IL-6, IL-12, and MCP-1, displaying notable pro-inflammatory effect. The surface markers of M2 macrophages are mainly CD163 and CD206, which can secrete anti-inflammatory factors such as IL-4, IL-10, transforming growth factor-β (TGF-β) and platelet-derived growth factor (PDGF), primarily playing anti-inflammatory and promoting tissue repair role.

### Role of Macrophages in Orthopedic Diseases

Macrophages are crucial in the pathogenesis of various musculoskeletal diseases such as osteoarthritis (OA) and osteoporosis (OP) (Xie et al., 2019; Muñoz et al., 2020; Tamaddon et al., 2020). Human CD14^+^ synovial macrophages can produce matrix metalloproteinases (MMPs), such as MMP-1 and MMP-13, which lyses type II collagen, worsen the inflammatory microenvironment of extracellular matrix (ECM), and accelerate the degeneration process (Wang and He, 2018). Synovial macrophages and monocyte-derived pro-inflammatory macrophages negatively regulate the chondrogenesis of bone marrow mesenchymal stem cells (MSCs) (Lepage et al., 2019). Inflammatory response in the synovium promote macrophages aggregation and synovial hyperplasia, ultimately leading to OA. Macrophages are extremely critical in maintaining osteogenic/osteoclast balance by influencing the secretion of inflammatory factors from osteoclasts (Srivastava et al., 2018). In inflammatory state, TNF-α, IL-1β and IL-6 promote osteoclast differentiation and bone resorption (Jung et al., 2019). Besides, macrophages are also closely involved in the regulation of rheumatism, rheumatoid arthritis, ankylosing spondylitis and femoral head necrosis.

Traditionally, the IVD was thought to be an immune privileged avascular organ (Stapels et al., 2016). However, Nakazawa et al. (2018) reported the existence of macrophages labeled with CCR7+, CD163+ and CD206+ in human IVD, and more and more scholars have focused on researching the role of macrophages in IVDD recently. Silva et al. (2019) discovered that many macrophages exist in degenerative IVD, and macrophages are often released into tissues through the circulatory system (Takeoka et al., 2020). Risbud and Shapiro (2014) confirmed that under inflammatory environment, NP secrete chemokine CCL2, CCL3 and CXCL10, which promote macrophages polarization, recruitment and migration, and produce more IL-1β and TNF-α, enhance the synthesis of MMPs, ultimately aggravate the IVDD. Zhang et al. (2016a) reported that the expressions of IL-17, CCL20 and CCR6 were markedly increased in the rat IVDD model, and it may be recruited into the degenerative IVD tissue through the CCL20/CCR6 system. The expression of IL-17 in the degenerative IVD tissue was notably correlated with CCL20 and CCR6. Chemokines and damaged tissue fragments lead to a large number of macrophages to recruit into damaged site, which further exacerbated the inflammatory microenvironment of IVD (Sainoh et al., 2015; Wiet et al., 2017; Torre et al., 2019).

### Effect of Macrophages on Low Back Pain by Acting on Intervertebral Disc

**FIGURE 2:**
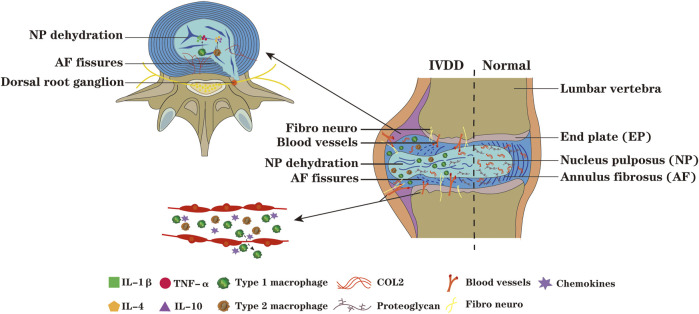
During the process of IVDD, the NP tissue protrude along the ruptured AF, nerves and blood vessels infiltrate and grow into IVD,and its adjacent tissues,and chemokines recruit macrophages from peripheral tissue of IVD and peripheral blood into IVD. The macrophages M1 polarization can secrete pro-inflammatory factors such as IL-1β and TNF-α, which aggravate the degeneration of IVD, while the macrophages M2 polarization can secrete anti-inflammatory factors such as IL-4 and IL-10. When the IVD is in a state of persistent inflammation, the degradation of ECM enhances; the inflammatory microenvironment promote the macrophages toward M1 polarization and secrete more pro-inflammatory factors, forming a vicious circle and inducing disc degeneration. The increased ingrowth of nerves into the IVD, and the continuous exposure of the IVD and DRG to inflammatory microenvironment further exacerbate IVDD.

IVDD is mainly characterized by IVD dehydration, ECM degradation, decreased proteoglycan content, collagen type transformation and AF rupture (Cazzanelli and Wuertz-Kozak, 2020), its exact mechanism remains to be elucidated (Murray et al., 2012; Zhang et al., 2016b). Due to the progressive degeneration process is often accompanied by increased levels of pro-inflammatory cytokines, IVDD is often considered as a chronic inflammatory state (Risbud and Shapiro, 2014; Cornejo et al., 2015). However, this inflammatory response is more likely to occur in response to tissue damage of the IVD rather than antigen specific inflammatory immune response. The traditional view is that immune cells have no effect on the pathophysiological process of IVD. Studies have reported that macrophages could recruit into the degenerative or herniated IVD region, infiltrate into closed NP (Shamji et al., 2010), which notably exacerbates IVDD (Figure 2).

Macrophages can promote the synthesis and release of inflammatory factors and aggravate IVDD (Wang et al., 2016; Peng et al., 2021). M1 macrophages-induced inflammatory effect act as a crucial role in IVDD (Zhang et al., 2021). M1 macrophages activate NP cells to secrete pro-inflammatory cytokines and chemokines, release proteolytic enzymes, break down the ECM components of the IVD, and exacerbate IVDD (Zhao et al., 2021). With the aggravation of IVDD, macrophages further infiltrate into the IVD. The secretion of inflammatory mediators tends to derive from the degenerative NP tissues (Willems et al., 2016). Nakazawa et al. (2018) detected the degenerative IVD and demonstrated that the expression of macrophages markers in damaged NP, AF and EP regions notably increased with the deterioration degree, and these markers were not found in healthy IVD. Long-term inflammation of the AF result in fissures, and in severe cases the NP protruded, which cause or aggravate LBP (Zhou et al., 2021). The literature documented (Nakazawa et al., 2018) that CCR7+, CD163+ and CD206+ cells in EP gradually increased with the deterioration, and the cell morphology and tissue structure exhibited obvious irregularities, supporting the hypothesis that exogenous macrophages infiltrate through the EP.

### Effect of Macrophages on Low Back Pain by Acting on NP

Macrophages infiltrating the NP may influence IVDD through the following pathways. On the one hand, the increased ECM degradation leads to the destruction of the IVD structure and the ingrowth of neovascularization, which promotes the more macrophages infiltration into the IVD. In the early stage of IVDD, the lysosomes in macrophages release degrading enzymes to decompose harmful substances phagocytized into cells, acting as an important role in local defense (Dongfeng et al., 2011; Zhang et al., 2016b). On the other hand, the inflammatory environment formed in the degenerative IVD will induce the accumulation of senescent cells, promote the infiltration of macrophages toward pro-inflammatory M1 polarization, and worsen IVD inflammatory microenvironment (Bisson et al., 2021). IL-4-induced macrophages to M2 polarization efficiently promote wound healing and tissues repair, but their pro-inflammatory and anti-infection abilities are weaker than those of M1 macrophages (Mosser and Edwards, 2008). Macrophages in or near IVD tissues can secrete pro-inflammatory factors such as IL-1β, TNF-α, IL-6 etc., which aggravate the inflammatory phenotype of NP. The exposed NP stimulates the recruitment of circulating monocytes into the NP tissues to differentiate into macrophages and secretes IFN-γ, which further promotes the migration of macrophages into the IVD (Shamji et al., 2010).

Dongfeng et al. (2011) confirmed that TNF-α and CD68 positive cells were highly expressed in the high-density area of IVD. The CD68 is a macrophage-specific antigen, and CD68 immunostaining positive cells were observed in the degenerated NP and AF. Macrophages, neutrophils, and T cells infiltrate the herniated and degenerated IVD following releasing chemokines from IVD cells (Risbud and Shapiro, 2014). Inflammatory factors infiltration into the IVD and those produced by the NP together contribute to IVD inflammatory microenvironment and aggravate IVDD (Guo et al., 2020; Hernandez et al., 2020; Qi et al., 2020). The expression of IL-1β, TNF-α, vascular endothelial growth factor (VEGF) and its receptor basic fibroblast growth factor (BFGF) are up-regulated in degenerative IVD, the catabolism of neovascularization is accelerated, and MMP synthesis is increased (Risbud and Shapiro, 2014). Yokozeki et al. (2021) documented that TGF-β can regulate resident macrophages in adult mice in addition to its self-regulatory effect, and age-related decline in TGF-β expression leads to decreased amount of macrophages in IVD, which is critical for tissue homeostasis and immune regulation. Hence, the excessively decrease in macrophages may result in imbalance homeostasis of IVD microenvironment, triggering or exacerbating LBP.

### Effect of Macrophages on Low Back Pain by Acting on AF

Due to the functional and structural integrity of normal IVD, nerves and blood vessels cannot grow into the interior of the IVD. When IVDD occurs, the degradation of ECM increases, and the AF (especially the outer AF) produces cracks. The fissure penetrates the AF and the interior of the IVD, creating a prerequisite for the migration and infiltration of macrophages into the IVD (Yamamoto et al., 2022). Kadow et al. (2015) confirmed that after AF tear, granulation tissue grows along the fissure from the outer AF to the inner AF and NP, and the vascularized AF and NP recruit macrophages to migrate into the IVD, generating more pro-inflammatory factors; above phenomenon eventually exacerbated inflammation of the IVD and promotes the occurrence and development of LBP.

The AF is affected by inflammatory factors secreted from macrophages, the synthesis of type II collagen and proteoglycan reduced, and production of tissue inhibition of metalloproteinase-1 (TIMP-1) increased, making the damaged IVD difficult to repair (Chu et al., 2018). Sainoh et al. (2015) confirmed that IL-6 and IL-6 receptor (IL-6R) levels peaked on the first day following IVD injury, IL-6/IL-6R positive cells in the AF and EP might be the macrophages infiltration into IVD. Following the AF injury, macrophages activation will trigger phagocytosis and pro-inflammatory reaction to remove necrotic cells and tissue fragments, chemokines and damaged tissue fragments will result in a large number of macrophages to recruit at the damaged region; during the process of elimination tissue fragments, the inflammatory microenvironment of the IVD will further deteriorated, which induce or exacerbate IVDD (Torre et al., 2019). Lin et al. (2020) reported that the expression of Tenomodulin (Tnmd) is markedly upregulated in the outer AF, and the loss of Tnmd promote IVD angiogenesis and macrophages infiltration, aggravate IVD inflammation and lead to LBP. Tnmd could efficiently inhibit angiogenesis and reduce inflammatory response induced by macrophages infiltration, which provides a new direction for the development of new drugs for preventing and treating LBP caused by IVDD.

### Effect of Macrophages on Low Back Pain by Acting on Endplate

The EP is composed of hyaline cartilage and subchondral bone. When the EP is damaged, the NP can directly expose to immune microenvironmental condition, producing many inflammatory mediators, M1 macrophages and activated T cells etc., resulting in further aggravation of EP injury and occurrence of LBP (Zheng et al., 2018). Ni et al. (2019) confirmed that during EP degeneration, osteoblast/osteoclast imbalance exacerbated porous structure in EP, stimulated sensory nerve fibers grew into EP and even the inside of IVD; the innervation level of Prostaglandin E2 (PGE2) and sensory nerve in porous EP increased, and PGE2 could activate its receptor EP4 in sensory nerve, and ultimately leads to spinal hyperalgesia. Multiple cavities formed in the EP as degeneration worsen, which may be largely attributed to inflammation mediated by macrophages (Rodriguez et al., 2012). M1 macrophages secrete TNF-α, IL-1β and IL-6 to promote the formation of osteoclasts, and M1 macrophages can also directly differentiate into osteoclasts (Yamaguchi et al., 2016; Dou et al., 2018). Chen et al. (2021) recently documented that during the co-culture of macrophages and bone MSCs (BMSCs), after the macrophages were polarized from M1 to M2, the expression of pro-inflammatory factors TNF-α, IL-1β and CCR7 in BMSCs notably decreased, while the expression of anti-inflammatory factors IL-4, IL-10 and CD206 were markedly increased, the expression of osteogenesis-related molecules were increased, and the alkaline phosphatase activity was enhanced. M1 and M2 macrophages have different effects on BMSCs osteogenic differentiation. M1 macrophages contribute to bone formation but do not promote matrix mineralization in the early stage of fracture repair, while M2 macrophages can promote matrix mineralization and MSCs osteogenic differentiation (Pajarinen et al., 2019). In conclusion, promotion of macrophages polarization to M2 is expected to efficiently repair the cavities in degenerative EP, inhibit the growth of nerves and blood vessels into IVD, and thus relieve LBP.

EP gradually calcifies during degeneration, increasing the risk of EP microfracture; promotion macrophages toward M2 polarization is expected to efficiently alleviate EP degeneration (Nagaraja et al., 2015; Noriega et al., 2017; Che-Nordin et al., 2018). EP degeneration often accompanied with Modic changes. The Modic changes refer to the bone marrow signal changes in MRI of vertebral EP and subchondral bone, which can predict the occurrence of LBP to a large extent. Modic changes are classified into three different types: Type I changes (low T1 and high T2 signals) are associated with EP tears and subchondral intramedullary vascular proliferation; Type II changes (high T1 and T2 signals), reflecting bone marrow steatosis; Type III changes (low T1 and T2 signals) mainly reflected as subchondral bone sclerosis. Type I Modic changes are more closely associated with LBP than other types. Dudli et al. (2016) confirmed that EP injury and autoimmunity are potential risk factors for Modic changes, and the number of protein gene product 9.5 (PGP9.5) nerve fibers and TNF-α positive cells in Type I and Type II changes in EP significantly increased. Yamamoto et al. (2022) reported that bone marrow derived macrophages (BMDMs) infiltrate the outer AF and EP in the degenerative IVD, and tissue-resident macrophages appear in the AF and NP. The macrophages are mainly M2 polarized, and M2a macrophages can result in tissues fibrosis and sclerosis, exacerbate the process of IVDD. As far as we known, BMDMs are the precursor cell of osteoclast, and the infiltration of BMDMs into EP are mainly M2 macrophages; the BMDMs in EP might enhance the EP sclerosis and eventually worsen IVDD.

### Macrophages Affect Low Back Pain Through Intervertebral Disc Adjacent Tissue

Macrophages affect LBP through the surrounding tissues of the vertebral body (Figure 2). The low back muscles can maintain the stability and function of the spine, muscle atrophy, increased myoelectric activity or muscle spasm will induce LBP (Hodges and Danneels, 2019). Vertebral facet joints can limit spinal hyperactivity, but with the aggravation of IVDD, facet joints are prone to degeneration (Perolat et al., 2018). When DRG is compressed by herniated disc and stimulated by inflammation, the symptoms of LBP will occur (Walker et al., 2014; Yu et al., 2020). James et al. (2018) discovered that macrophages and TNF-α expression obviously increased in multifidus muscle in degenerative disc segment of sheep, and M1 macrophages secreted TNF-α in paravertebral multifidus muscle. The number of M1 macrophages increased after muscle injury, inducing satellite cell proliferation and fibroblast progenitor cell apoptosis, promoting myoblast fusion. Slow fiber loss in multifidus induces macrophages M1 polarization. Multifidus structure changes in the subacute stage express a variety of inflammatory factors, facilitate macrophages migration into the injured site, aggravate paravertebral muscle inflammation and bring about LBP (Hodges et al., 2014; Hodges et al., 2015). When Netzer et al. (2016) detected facet joints in patients with degenerative lumbar spinal stenosis, they found extensive *de novo* bone formation and inflammatory cell infiltration into subchondral bone marrow cavity, characterized by high abundance of macrophages. In the muscles, DRG, and facet joints around the IVD, the accumulation of macrophages caused by mechanical load, chronic injury and other reasons; macrophages combined with factors such as pro-inflammatory factors, neural factors, and vascular nerve ingrowth, together triggering or exacerbating LBP.

### Macrophages Affect Low Back Pain Through Muscle

The macrophages M1 polarization lead to the transformation of muscle from slow muscle fiber to fast muscle fiber, resulting in lactic acid accumulation and macrophage-dominated inflammatory response. Macrophages secrete inflammatory factors that engulf necrotic muscles, reduce contraction potential, irritate hyperalgesia, and aggravate LBP (James et al., 2018). Villalta et al. (2009) reported that skeletal muscle of MDX transgenic mice contained more pro-inflammatory, typically activated M1 macrophages, which lysed muscle through the synthesis and release of nitric oxide synthase *in vitro*. The two different polarized forms of macrophages maintain the muscle lysis-fibrosis balance during muscle remodeling and avoid the occurrence of LBP. Muscle fatigue down-regulated the PH and activates acid-sensitive ion channel 3 (ASIC3) in resident macrophages, which release pain-related chemicals and cause hyperalgesia. Gong et al. (2016); Gregory et al. (2016) reported that the gene deletion of ASIC3 and pharmacological inhibition of APETx2 on ASIC3 in mice can prevent hyperalgesia induced by muscle fatigue. Exercise/acupuncture therapy for muscle pain can effectively promote macrophages toward anti-inflammatory M2 polarization and the release of anti-inflammatory factor IL-10. Fatigue metabolites can activate macrophages to locally release IL-10 and promote the macrophages toward M2 polarization, which efficiently relieve LBP (Leung et al., 2016). Novak et al. (2014) confirmed that the transition of the muscle macrophages population from an early heterogenous and hybrid phenotype to the subsequent inactivation state was due to IL-10 producted by macrophages.

James et al. (2016) verified that although MSCs therapy prevent fat infiltration and fibrosis of multifidus when IVDD occurs, it cannot adequately prevent muscle inflammation and muscle fiber type transformation; it is necessary to combine the induction of macrophages toward M2 polarization in order to relieve LBP. Du et al. (2017) reported that in the wild-type mouse muscle injury model, macrophages in the injured muscles secrete A Disintegrin and Metalloproteinase with Thrombospondin Motif 1 (ADAMTS1) in large amounts, and ADAMTS1 activate muscle stem cells by inhibiting Notch1 signaling pathway, promote regeneration of injured muscles, and obviously relieve LBP. Zhao et al. (2016) documented that in mouse models of acute skeletal muscle injury, deletion of chemokine receptor CX3C chemokine receptor 1 (CX3CR1) in macrophages has not affect the number of monocytes/macrophages in the injured muscles, but impaired the phagocytic function of macrophages, reduced the expression of insulin-like growth factor-1 (IGF-1) in macrophages, and delayed the repair of damaged muscles. Muscles repair depends on macrophage-mediated inflammation to some extent, and the influence of CX3CR1 on macrophage function will lead to persistent pain. Varga et al. (2016) displayed that peroxisome proliferator-activated receptor γ (PPARγ) regulates the expression of growth differentiation factor 3 (GDF3), a member of the TGF-β family, and is extremely important for muscle repair and regeneration. Macrophages are the main source of GDF3 in damaged tissues. Macrophage-mediated muscle regeneration is largely correlated with the PPARγ-GDF3 pathway, which provides a new idea for repairing muscle injury to alleviate LBP.

### Macrophages Affect Low Back Pain Through Dorsal Root Ganglion

Macrophage-mediated inflammation and immune response play an important role in neurogenic pain (Scholz and Woolf, 2007). Kim and Moalem-Taylor (2011) discovered that the infiltration of macrophages, neutrophils, and dendritic cells in the injured sciatic nerve and ipsi-lateral DRG markedly increased, and the density of macrophages at the injured site increased up to 7 times, inducing or aggravating pain. The acute infiltration of neutrophils to injured nerve reaches its peak within a few hours after injury, and neutrophils release can sensitize a variety of chemokines, notably promoting the recruitment and activation of macrophages (Kumar and Sharma, 2010). Nadeau et al. (2011) reported that sciatic nerve injury induces the production of IL-1β and TNF-α, resulting in neutrophils and M1 macrophages infiltrating the distal nerve. Compared with wild-type mice, neutrophils and M1 macrophages were significantly reduced in IL-1R1 and TNFR1 deficient mice, which are critical for inducing macrophages to infiltrate into the damaged nerve.

Macrophages can affect nerve regeneration by regulating nerve growth factor (NGF), and the recovery of nerve function depends on the expression of IL-1β and TNF-α to some extent; and thus, severe depletion of macrophages hinder the axons regeneration in damaged nerves (Barrette et al., 2008). Increased levels of NGF, brain-derived neurotrophic factor (BDNF) and inflammation promote the nerve fibers of DRG growth into the AF and NP, enhance the sensitivity of pain cationic channel, and bring about LBP (Risbud and Shapiro, 2014; Kadow et al., 2015). There are resident macrophages expressing CD163 in the DRG, as well as macrophages containing CD68^+^ lysosomes and major histocompatibility complex II (McLachlan and Hu, 2014). Within days to weeks of sciatic nerve injury, activated macrophages and T lymphocytes migrate from lymph nodes and spleen to the injured region, and selectively migrate and recruit to the damaged DRG (Kim and Moalem-Taylor, 2011; Schmid et al., 2013). Immune cells, which are rich in ganglia, release the pro-inflammatory factor TNF-α, has been reported to directly increase neuronal firing rates (Ibeakanma and Vanner, 2010) or modify gene expression (Grisanti et al., 2011). Together, the macrophage-mediated inflammation and its positive feedback activation further exacerbate DRG damage, leadind to spinal hyperalgesia and LBP.

### Macrophages Affect Low Bacl Pain Through Facet Joint

Studies on the degenerative joint capsule demonstrated that inflammatory pain mediators and chondrodegrading enzymes were highly upregulated (Netzer et al., 2016); the histopathological features of subchondral bone were infiltration of macrophages and enhancement of *de novo* bone formation in bone marrow. Kim et al. (2015) confirmed the infiltration of CD11d positive cells (macrophages) and increased production of inflammatory cytokines in the facet joint and capsule tissues with severe degeneration. Compared with normal facet joint, the levels of proinflammatory factors and chondrodegrading enzymes in degenerative facet joints are increased, and macrophages play an important role in the structural and functional changes of facet joints. Not only pro-inflammatory cytokines and chondrodegrading enzymes increased, but also anti-inflammatory cytokines and chondrodegrading enzyme inhibitors IL-10, IL-13, TIMP-2 and TIMP-3 were also upregulated, suggesting that this might be a tissue repair reaction (Kim et al., 2015). Migration and aggregation of macrophages may be an pivotal causative factor of LBP attributed to facet joint degeneration (Perolat et al., 2018). After facet joint injury, synovial macrophages secrete pro-inflammatory signaling molecules, such as Alarmins (van den Bosch et al., 2016), IL-1 and TNF-α (Manferdini et al., 2016), meanwhile the production of MMPs and inflammatory factors increases, which aggravate facet joint degeneration. Spinal degenerative diseases can be accompanied by facet joint-derived LBP, middle-aged and elderly patients as well as patients with spinal deformity should be paid more attention.

### Macrophages Affect Low Back Pain Through Other Tissue

In the fat and connective tissue adjacent to IVD, macrophage-mediated inflammation can result in LBP. James et al. (2018) used immunofluorescence to localize macrophages in sheep IVDD model; the results demonstrated that TNF expression in adipose and connective tissue in multifidus muscle was markedly increased, which was positively correlated with pro-inflammatory M1 macrophages. James et al. (2021) evaluated the multifidus muscle and subcutaneous fat in 24 patients with disc disorders, and found that TNF expression was notably increased in patients with high infiltration of fat in multifidus muscle, suggesting that inflammatory factors might be an crucial driver of fat formation. It has been reported (Dalmas et al., 2011) that adipose tissue inflammation is largely attributed to the proinflammatory action of macrophages from bone marrow derived white adipose tissue (WAT). The WAT macrophages localize to dead adipose cells and form coronal structures (CLS) that fuse into pro-inflammatory multinucleated giant cells, and these CLS account for more than 90% of infiltrating macrophages (Dalmas et al., 2011). Although some reports (Chung et al., 2017; Nøhr et al., 2017) suggested that macrophage-induced inflammation obviously inhibit fat browning, there still remains controversial. Yang et al. (2018) reported that spinal disc puncture modeling in rats distinctly increase the number of microglias in the lumbar spinal cord, and the expression of colony stimulating factor 1 receptor (CSF1R) in microglias is increased, and the CSF1/CSF1R signaling pathway contributes to microglia activation, which further enhance the neurosensitivity of the central nervous system and lead to LBP.

### Targeted Regulation of Macrophages to Relieve Low Back Pain

**FIGURE 3:**
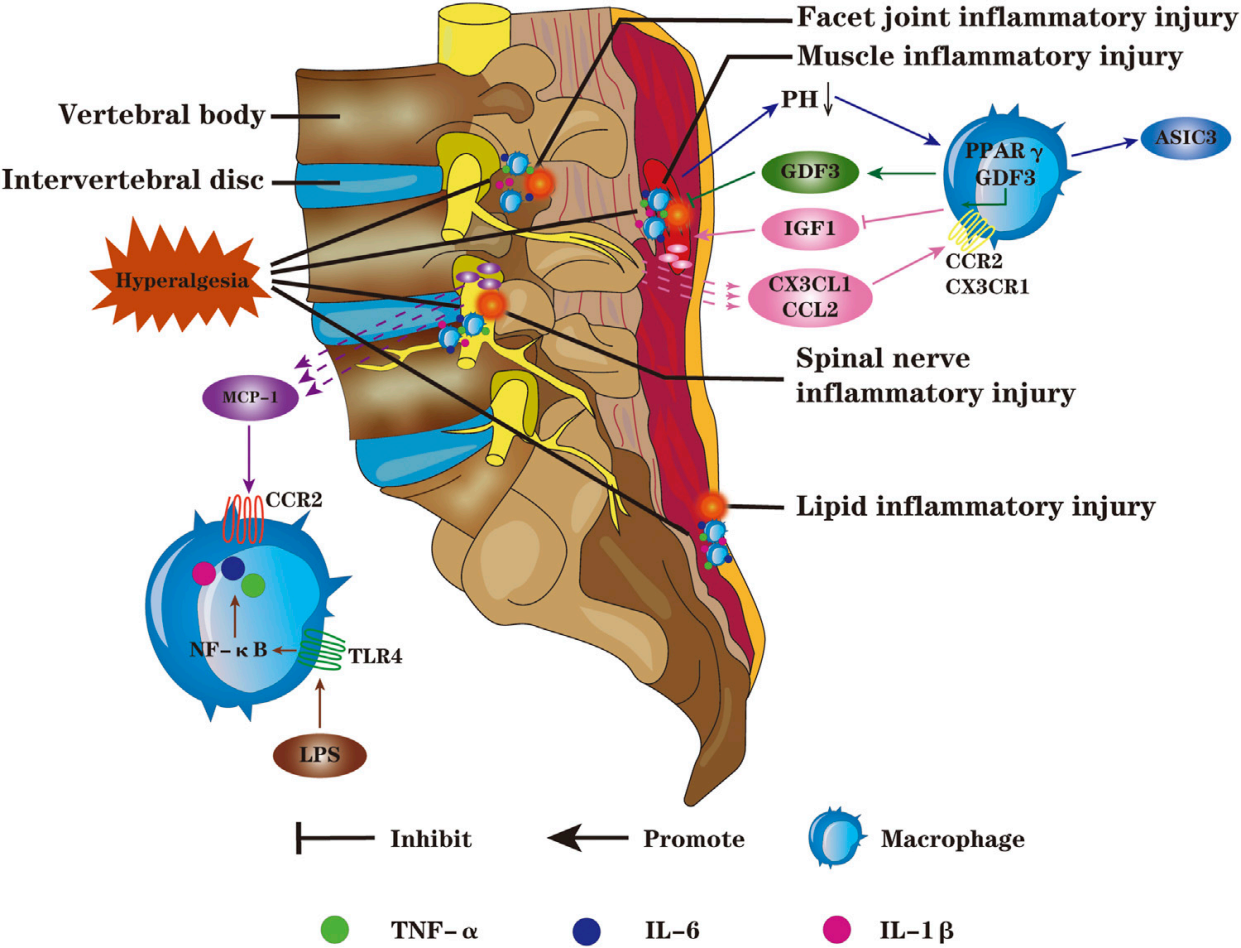
Macrophages can chemotaxis and migrate to adjacent tissues of IVD such as paraspinal muscles, DRG, facet joints and fat; and then synthesize more pro-inflammatory factors such as TNF-α, IL-1β and IL-6, resulting in spinal cord hyperalgesia, inducing or exacerbating LBP. The macrophages toward M1 polarization will lead to the accumulation of lactic acid in muscle, and the decrease of PH value can up-regulate the expression of ASIC3. Macrophages infiltrating into the damaged DRG promote the secretion of inflammatory mediators and induce neurogenic pain; macrophages can infiltrate the facet joints, aggravate the inflammation and degeneration of the facet joints, and cause LBP. Macrophages are the main source of GDF3 in damaged tissues, and PPARY in macrophages can promote tissue repair and regeneration by regulating the expression of GDF3. Chemokines CX3CL1 and CCL2 recruit macrophages and bind to CX3CR1 and CCR on macrophages,respectively, inhibiting the release of IGF1 and aggravating the inflammatory response; the chemokine MCP-1 could bind to CCR2, inducing and aggravating inflammation. The LPS acting on TLR4 in macrophages will lead to increased release of pro-inflammatory factors through the NF-KB signaling pathway.

Activation of macrophages can induce or exacerbate IVDD; if the pathogenic factors are not removed in time, IVDD will gradually worsen to irreversible pathological state. Under the condition of macrophage-mediated inflammation, tissue damage and structural remodeling of adjacent IVD tissues also induce or aggravate LBP (Figure 3). Rajan et al. (2013) displayed that the expression of toll-like receptor 4 (TLR4) in NP, AF and EP cells is controlled by its ligand. When ligand binding to TLR4, it will result in upregulation of inflammatory mediators, such as TNF-α, IL-1β, IL-6 and macrophage migration inhibitory factor, damaging the normal IVD structure. Targeted regulation of TLR4 expression is expected to effectively relieve LBP caused by IVDD. Wu et al. (2017) discovered that the levels of pro-inflammatory factors such as IFN-γ, TNF-α and IL-1β were notably increased in facet joints; the injection of platelet rich plasma exerts an excellent anti-inflammatory effect by down-regulation inflammatory mediators in synovium cells and chondrocytes, and the effect is better than cortisol injection. Ohtori et al. (2012a); Ohtori et al. (2012b) confirmed that, in patients with lumbar spinal stenosis, TNF-α activation in DRG is closely related to pain generation and maintenance. Spinal epidural use of TNF-α inhibitors or anti-IL-6γ monoclonal antibodies markedly alleviates sciatica. In a 3-years follow-up of randomised double-blind trial, Genevay reported similar results (Genevay et al., 2012), effective relief in leg pain was observed following subcutaneous injection human anti-TNF-α antibodies (Table 2).

**TABLE 2:** Molecular targets related to treatment of LBP by regulating macrophages-mediated inflammation.

	Therapeutic mechanism and target	References
IL-1β	CCR antagonist reduce the IL-1β-mediated disc inflammation;	Chou et al. (2020)
	MCP-1 expression in macrophages increased with IL-1β, downregulation of IL-1β reduce macrophage recruitment;	Kawakubo et al. (2020)
	Macrophage migration inhibitory factor controls the expression of NLRP3, induced IL-1β activation;	Kang et al. (2019)
	Upregulation of silent information regulator 2 homolog 1 by tyrosol suppress the IL-1β-mediated inflammation	Qi et al. (2020)
IL-6	Inhibiting IL-6/IL-6R expression through gp130/JAK-STAT3 signaling pathway, relieve LBP induced by inflammation;	Torre et al. (2019)
	Epidural injection of anti-IL-6 receptor monoclonal antibody relieve radicular pain;	Ohtori et al. (2012b)
	Nanoparticle FT-C60 bind to FPR-1 on macrophages can notably attenuate the expression of IL-1, IL-6 and TNF-α;	Xiao et al. (2019)
	Intradiscal injection of the IL-6 inhibitor alleviate LBP	Sainoh et al. (2015)
TNF-α	In IVD injury model, TNF-α stimulates CCL2-mediated recruitment of macrophages;	Nakawaki et al. (2020)
	Epidural application of TNF-α inhibitor etanercept can effectively relieve pain;	Ohtori et al. (2012a)
	RhoA rescued IVD cells from TNFα-induced inflammation and mechanobiological disruption	Hernandez et al. (2020)
IL-4	shDNMT1 notably increased IL-4, induced M2 macrophages polarization	Hou et al. (2020)
IL-10	Physical activity increases the percentage of regulatory macrophages in muscle which can relieve chronic musculoskeletal pain, and that IL-10 is an essential mediator;	Leung et al. (2016)
	IL-10 regulate and relieve the muscular dystrophy by reducing M1 macrophages activation	Villalta et al. (2011)
PGE2	Inhibiting of the PGE2/EP4 pathway efficiently improves spinal hyperalgesia	Ni et al. (2019)
ASIC3	The decreases in pH induce release of inflammatory cytokines possibly through activation of ASIC3 on macrophages, and eventually result in hyperalgesia;	Gong et al. (2016)
	Injecting the ASIC3 antagonist ApeTx2 into the muscle efficiently inhibit pain sensitivity;	Gregory et al. (2016)
	Lactate regulates ROS generation through ASIC3, promoting IL-1β release	Zhao et al. (2021)
CCR	CCR antagonist reduce the IL-1β-mediated disc inflammation;	Chou et al. (2020)
	IL-1β notably upregulate CCR7 expression and increase production of IL-6 on macrophages;	Silva et al. (2019)
	TNF-α and IL-1β dependent CCL, induce macropahges migration through CCR activation	Wang et al. (2013)
TLR4	TLR4 relieves pain by inhibiting the expression of TNF and IL-1β activated by the NF-κB signaling pathway;	Wu et al. (2010)
	Resistin binds to TLR4 in NP cells, result in macrophages infiltration;	Li et al. (2017)
	Following administration of TLR4 inhibitor, TNF-α and IL-1β markedly decreased, while IL-10 increased	Kuang et al (2012)
CSF1/CSF1R	Targeted inhibition of CSF1/CSF1R signaling pathway to suppress microglia activation and related inflammation might be a promising strategy to alleviate LBP	Yang et al. (2018)

### Regulation of Macrophages to Relieve Low Back Pain Caused by Intervertebral Disc Degeneration

Macrophages infiltrate into the IVD and act as critical role in initiating IVD inflammation and immune response (Chou et al., 2020; Kawakubo et al., 2020). Targeted regulation of macrophages polarization helps to the development of tissue repair and relief of LBP. The migration inhibitors not only play an important role in macrophages migration, but also positively regulate other pro-inflammatory factors (Kang and Bucala, 2019). During disc degeneration, macrophages can indirectly participate in inflammation by recruiting monocytes through blood, therefore inhibition of monocyte recruitment is an effective strategy to prevent inflammation and IVDD (Kawakubo et al., 2020).

Hasvik et al. (2019) illustrated that many miRs affect the expression of disc degeneration diseases related genes, for instance, miR-17 simultaneously regulates the synthesis of signal regulator protein α (SIRPα) in NP cells and its mediated activation of macrophages, increasing TNF production. The miR-17 play a pro-inflammatory effect by stimulating the expression of TNF, and regulation of miR-17 might be an effective strategy for the prevention and treatment of LBP. Nakawaki et al. (2020) reported that TNF-α facilitates the synthesis of CCL2 in disc degeneration, which in turn mediates the recruitment and macrophages toward M1 polarization, accelerating IVDD process. Wang et al. (2013) analyzed the degeneration of human NP and discovered that it could induce the expression of CCL3 stimulated by p38 mitogen-activated protein kinase (MAPK) under proinflammatory factors; CCL3 binds to its receptor CCR1 and exerts a pivotal role in macrophages migration, which was positively correlated with IVDD. Li et al. (2017) demonstrated that the expression of CCL4 was increased in human degenerative NP tissues, which notably induced macrophages migration to NP by binding CCR1. Further studies have shown that the binding of statin to its receptor and TLR4 in the NP promoted the expression of CCL4 through P38 MAPK and nuclear factor kappa B (NF-κB) pathway. Xiong et al., (2012) confirmed that migration inhibitors in human degenerative IVD may interfere with the ability of CEP derived stem cells (CESCs) to migrate into inflammatory region. Hence, restore the migration function of CESCs is able to promote the regeneration and repair of degenerative IVD.

Hou et al. (2020) *in situ* injected adeno-associated virus carrying DNA methyltransferase 1 (DNMT1) shRNA into macrophage-specific CD68 promoter in a mouse model of IVDD, they found that shDNMT1 efficiently attenuated the levels of TNF-α, IL-1β and IL-6, promoted the anti-inflammatory factor IL-4 and IL-10 expression, and led to macrophages toward M2 polarization. Xiao et al. (2019) synthesized a novel formyl peptide receptor-1 (FPR-1) coupling targeting C60 nanoparticleft-C60 (FTC60), which has excellent targeting ability to the highly expressed FPR-1 receptor on macrophages. After binding with macrophages, FTC60 exerts a strong anti-inflammatory effect and remarkedly reduces the expression of IL-6, IL-1, TNF- α and COX-2. Inflammation and immune response are double-edged swords, mild inflammation is beneficial to tissue repair and remodeling. Analgesic treatment should be aimed at inhibiting excessive activation of inflammation rather than completely blocking inflammatory response. The specific strategy of regulating macrophages polarization to intervene inflammation and immune response, and then to relieve or even block LBP needs further study (Ellis and Bennett, 2013).

### Regulation of Macrophage-Mediated Inflammation and Immune Reponses in Muscles and Other Tissues to Relieve Low Back Pain

Villalta et al. (2011) confirmed that IL-10 inhibit macrophages toward M1 polarization in mouse muscle, induce the activation of M2c macrophages, promote muscle repair and regeneration, and regulate the balance between macrophages M1/M2c polarization can effectively treat muscular dystrophy. One study (Ruffell et al., 2009) investigated the role of M2 macrophages in the repair of muscle injury in mice and discovered that deletion of two binding sites in the cAMP response element binding protein-CCAAT enhancer binding protein β (CREB-C/EBPβ) pathway blocks the expression of M2 macrophage-associated phenotypic molecules. CREB-mediated elevated of C/EBPβ expression promotes M2 macrophage-related phenotypic molecular and muscle regeneration, accelerating muscle remodeling and relieving pain. Scholz and Woolf (2007) reported that CX3C chemokine ligand 1 (CX3CL1) and CCL2 released by DRG neurons after nerve injury leads to aggregation of macrophages, which can release TNF acting on TNFR1-P38-MAPK signaling pathway to enhance tetrodotoxin-resistant voltage-gated sodium channel, resulting in spinal hyperalgesia and LBP. Sato et al. (2015) evaluated the regulatory effect of anti-NF-κB receptor activator ligand antibody on sensory nerves in inflammatory areas, and displayed that receptor activator of NF-κB ligand (RANKL) expression was elevated in animal models of pain or disc herniation. Anti-RANKL not only inhibited the expression of pain-related neural peptide calcitonin gene-related peptide in DRG neurons, but also reduced the level of inflammatory factors in IVD. RANKL may be an effective target molecule for the treatment of discogenic LBP.

Jeon et al. (2009) reported that a variety of molecules are involved in the regulation of pain induced by nerve injury, such as MCP-1 and its receptor CCR2 in DRG. After nerve injury, the expression of MCP-1 in DRG reaches its peak and then declines before the complete onset of pain hypersensitivity; inhibition of MCP-1 efficiently relieved DRG-induced pain. Activation of neuronal TLR pathway regulates the macrophages polarization near DRG by generating CCL2 chemokine in nociceptors (Liu et al., 2012; Liu et al., 2014). Kuang et al. (2012) confirmed that TLRs targeting DRG directly regulate the expression of IFN-γ, IL-1β and TNF-α, and inhibition of TLR reduce the inflammatory factors expression and improve local inflammatory microenvironment of DRG. Targeted treatment of DRG is the focus of future research. Wu et al. (2010) documented that TLR4 antagonists and siRNA-TLR4 effectively inhibit the activation of NF-κB and production of TNF and IL-1β in rat models, alleviating mechanical pain and heat-sensitive pain caused by chronic contractile injury. The inhibition of TLR4 is a promising strategy for treatment of neurogenic pain. Wang et al. (2018) discovered that injection of low concentration Ozone obviously reduce the expression of pro-inflammatory factors, and therefore alleviate mechanical pain in rats with chronic radiculopathy. The cAMP, cGMP and NF-κB are downstream signaling molecules of phosphodiesterase. Low concentration of Ozone inhibits inflammatory response by regulating pDE2A-cAMP/cGMP-NF-κB/P65 signaling pathway, markedly relieving pain symptoms. Ozone stimulates macrophages to phagocytose and absorb the protrusion to keep the NP rejuvenated in the intervertebral space. Ozone also promote the macrophages from M1 to M2 polarization (Erario et al., 2021), which can be used as an effective treatment for LBP.

### Limitation

Although the research on the pathogenesis and prevention of LBP has been progressing, there are still some limitations. As most of the data reported so far are from high-income countries, it is unclear whether they apply to low and middle-income countries. There is a “network-like” interactive regulation between macrophages and inflammatory chemokines, it is difficult to precisely regulate a certain factor. The different macrophages polarization and the dynamics of their chemotactic aggregation and distribution in degenerated IVD is difficult to trace, so transgenic animals or near-infrared fluorescent macrophages probe *in vivo* tracing technology are needed. In addition to conventional treatment, regenerative medicine technology has great prospects, however, most of the current research is in the basic experimental stage, and many anti-inflammatory molecules or new drugs reported through cell experiments or animal models have not been used in clinic. Before the new targeted anti-inflammatory drugs discovered in basic research can be used in clinical treatment of patients with LBP, further research is needed to evaluate their safety and effectiveness.

## Conclusion

Macrophages can migrate into the IVD under the action of a series of inflammatory and chemokinetic factors, and play the role of triggering and cascading inflammatory and immune responses, inducing and aggravating IVDD and LBP. Meanwhile, macrophages induce inflammation and immune responses in tissues adjacent to the IVD, such as DRG, muscle, and facet joint, leading to spinal hyperalgesia and exacerbating LBP. Using specific drugs or tissue engineering to intervene macrophages and promote their transformation into anti-inflammatory M2 polarization is expected to provide a new strategy for effective prevention and treatment of LBP. It is of great significance to further deepen the basic research on the pathogenesis and treatment of macrophages-mediated LBP, which is expected to provide an exact drug target for the clinical treatment of IVDD and LBP.
